# Prospective patterns of modifiable health risk behaviors and the utilization of healthcare services in the “Health Workers Cohort Study” in Mexico

**DOI:** 10.1371/journal.pone.0208172

**Published:** 2018-12-06

**Authors:** Fernando Trujillo-Olea, Julián Alfredo Fernández-Niño, Jorge Salmerón, Katia Gallegos-Carrillo

**Affiliations:** 1 Hospital General Regional/MF No. 1. Cuernavaca, Instituto Mexicano del Seguro Social, Cuernavaca, México; 2 Departamento de Salud Pública, Universidad del Norte, Barranquilla, Colombia; 3 Unidad Académica de Investigación Epidemiológica, Universidad Nacional Autónoma de México, Ciudad de México, México; 4 Unidad de Investigación Epidemiológica y en Servicios de Salud, Morelos, Instituto Mexicano del Seguro Social, Cuernavaca, México; Seoul National University College of Medicine, REPUBLIC OF KOREA

## Abstract

**Introduction:**

We still lack information about how changes in modifiable health risk behaviors influence the utilization of healthcare services. This study assesses the relationships between prospective patterns of modifiable health risk behaviors and the utilization of healthcare services.

**Material and methods:**

This was a prospective study among men and women participants in the Health Workers Cohort Study, aged 18 years and older. The following data about modifiable health risk behaviors was collected in two waves of the study (2004–2006 and 2010–2012): 1) physical activity, b) consumption of fruit and vegetables, 3) alcohol, and 4) tobacco consumption, to determine the association between the utilization of healthcare services after 6 years of follow-up (period 2010–2012). Information was collected through self-administered questionnaires; clinical and anthropometric variables were measured following standard procedures. Analyses were conducted using zero-inflated negative binomial regression models.

**Results:**

Participants with a pattern of consumption of < 3 portions of fruits and vegetables per day (p = 0.035) and did not meet recommended levels of PA (p = 0.013) during the two waves of the study had fewer preventative consultations; those who quit smoking reported a higher frequency of preventative consultations (p = 0.021) and more visits with a medical specialist (p = 0.048). Participants who reduced alcohol consumption to low or completely stopped its consumption reported fewer visits to the general physician (p = 0.031), p < 0.05.

**Conclusions:**

Changes in prospective patterns of modifiable health risk behaviors influenced the use of healthcare services after 6 years of follow-up. Findings in this study could be useful to determine possible demands of healthcare services among populations with specific modifiable health risk behaviors.

## Introduction

Non-communicable diseases (NCD) are the main causes of disability adjusted life years worldwide [1]. Lifestyles consisting of unhealthy diets, physical inactivity, and alcohol and tobacco consumption are major contributors to the burden of NCD [2]. Due to the complexity and challenges associated with the prevention and treatment of NCDs through the change of unhealthy lifestyles, programs and interventions demand inter-sectorial actions, lead predominantly by guidance and assistance from the healthcare sector [2–4].

The WHO has also defined actions for health promotion in relation to four modifiable lifestyle factors: diet, physical activity [5], tobacco consumption [6] and alcohol consumption [7]. In the strategies designed by the WHO, the health system plays an important role in helping people lead healthier lifestyles (utilization of healthcare services***→*** MHRB) [8, 9]. However, the ways in which healthy or unhealthy lifestyles determine the utilization of healthcare services have only been studied preliminarily (MHRB***→*** utilization of healthcare services) [10–14]

Multiple determinants of the utilization of healthcare services have been identified, such as the modifiable health risk behaviors (MHRB), because those are factors which can explain the use of services due their influence on health status [15]. In addition to actions that promote health, other factors called social determinants are present [16] and may be involved as intermediaries in the relationship between lifestyles and use of services, or be common to both (Social determinants***→***MHRB***→***Health Status***→***utilization of healthcare services) [15, 17].

When talking about tobacco consumption and the utilization of healthcare services, Wacker, et al. reported that smokers have a lower probability of seeking medical consultations and physical therapy [13], and former smokers have a higher probability of treatments and hospitalization, rehabilitation and the use of medications [13]. One study concluded that participation in exercise programs decreased the use of a variety of healthcare services [12]. Another study found that the consumption of alcohol is associated with a lower utilization of healthcare services [14]. Lin and Cheng, reported a lower probability of using outpatient services when participants’ lifestyles included a healthy diet where meat, grain, fruit or milk are consumed on a daily basis [11]. Lastly, Feng et al., showed that persons with multiple unhealthy lifestyles are less likely to see a general practice physician during a 12-month period [10]. Nevertheless, the evidence about how change in modifiable health risk behaviors would influence the utilization of healthcare services is still lacking. The Mexican Institute of Social Security (IMSS, Spanish acronym), is a part of the Mexican health system and the main social security institution in Mexico, providing health insurance and a comprehensive package of benefits to its affiliated population including daycare, sports facilities, disability and life insurance and pensions. This includes approximately 50% of the country’s population (63,480,327 at 2016). On a typical day in the institute, 479,000 medical consultations are provided; 326,000 are only for primary health services, including both preventative services and treatment among its 2,000 health facilities around the country [18].

We must determine if changes in the modifiable health risks behaviors in the population influence the utilization of healthcare services. This is particularly important since in recent years a large portion of the expenditures for healthcare has been seen by portions of the population who are suffering the consequences of unhealthy lifestyles. Given the disease burden that the MHRB represent, there is no evidence to date regarding how changes in modifiable health risk behaviors influence the utilization of health services, particularly in middle income countries such as Mexico, which is experiencing a high burden of disease associated with health conditions such as obesity. Mexico has the second highest rate of obesity worldwide, and that is largely attributable to modifiable health risk behaviors in the Mexican population.

The objective of this study is to identify if prospective patterns of modifiable health risk behaviors are associated with the utilization of healthcare services after a six-year period among the participants of the Health Workers Cohort Study (HWCS) in Mexico.

## Methods

### Data

Analysis using the databases from the HWCS, this was a prospective study aimed to analyze the relationship between lifestyle factors and chronic diseases [19]. This study is a dynamic and open cohort study where the information was collected in three waves. Recruitment was voluntary and was carried out using an advertisement distributed within the health care institution. All of the health workers at the Mexican Social Security Institute (IMSS) in Morelos and their families were invited to participate. IMSS, is a public social security institute created in 1943 As of December 2014, 59.1% of the Mexican population was enrolled in this institute, that is, 71,151,867 enrollees. During this same year, the state of Morelos had 801,763 enrollees. Methodological issues and design of the study have been previously published [19].

The information about health services, modifiable health risk behaviors and socio-demographic variables was collected through a self-administered questionnaire. The information used by the present analysis included: use of health services, socio-demographic characteristics, records about health conditions and diseases, diet, physical activity, tobacco consumption and alcohol consumption. Clinical and anthropometric measurements were also obtained; weight, height and blood pressure; details described further below.

For the present study, we considered the information of the follow-up measurements collected during the second (2004–2006) and third waves (2010–2012) of the HWCS. According to the criteria, 80 participants were excluded from the initial sample (n = 1174) for being under 18 years old and 396 participants were excluded for having incomplete information related to the variables of interest in one or both waves, lifestyles n = 117, health services utilization n = 91 and co-variables n = 188. Therefore, the final analytical sample included 698 subjects.

### Dependent variables

Information was obtained about the utilization of the following healthcare services during the last 12 months prior to the data collection: visits to a family or general practice physician, medical specialist, nutritionist, psychologist, dentist, emergency room services, preventive medicine consultations, laboratory services, lab test, educational health sessions, health promotion activities and hospitalization. Based on the classification of health services proposed by Frenk, J. [20], the outcome variables proposed were: 1) preventative services (preventative medicine) and three therapeutic-diagnostic services (2) family/general practice, 3) medical specialist, 4) emergency room services). These variables were treated as count variables. Information about the use of healthcare services was available only during the last stage of the study (2010–2012).

#### Independent variables

**Fig 1:**
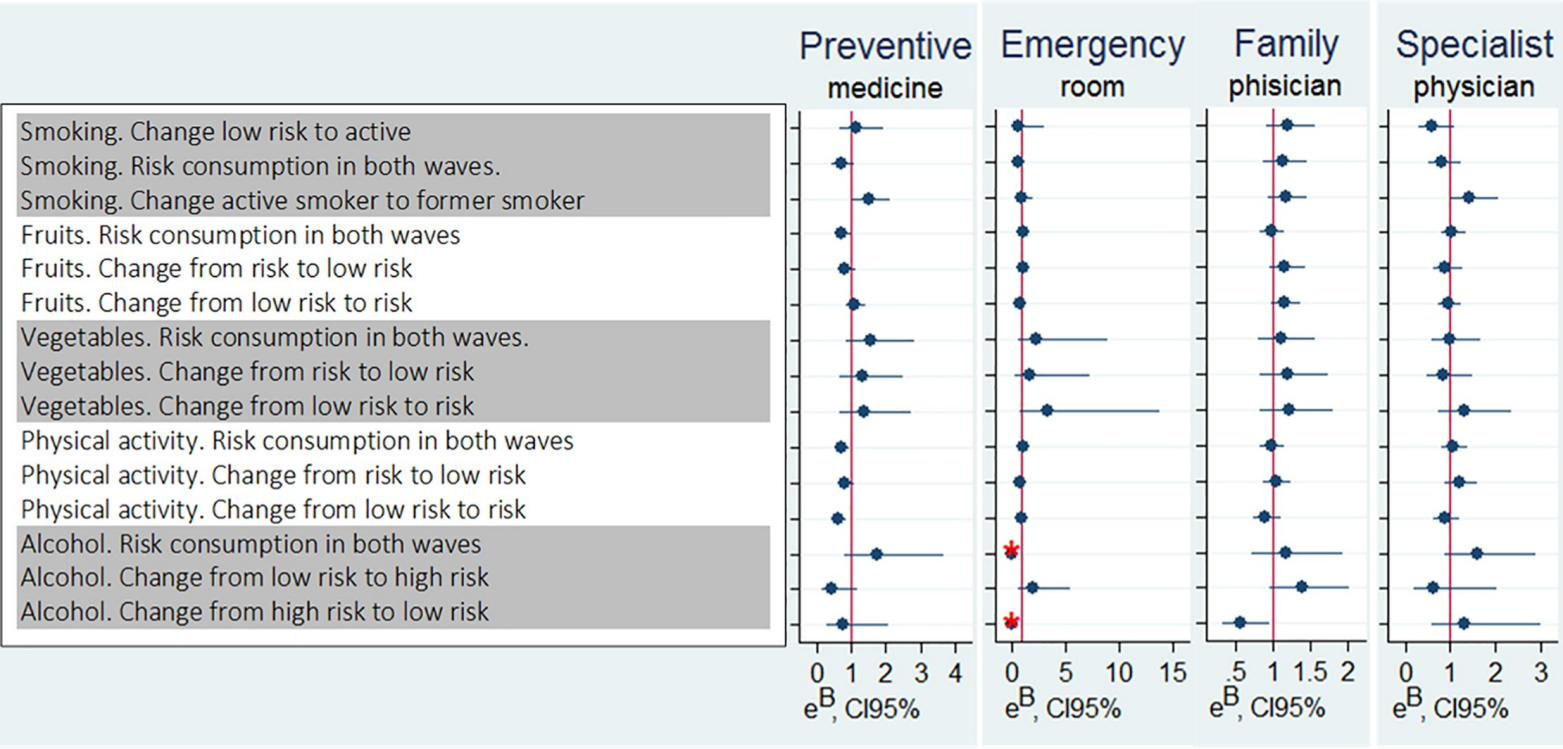
Graphic of the beta exponential (e^B^) and 95% confidence interval for the main explanatory variables of the use of four health services, in the Health Workers Cohort Study, Morelos, 2004–2010. Models adjusted for the following variables: tobacco consumption, consumption of fruits, consumption of vegetables, alcohol consumption, leisure time physical activity, chronic diseases, surgeries or injuries, ongoing consumption of medications, hyperuricemia, hyperlipidemia, hyperglycemia, chronic kidney disease, anemia, and high blood pressure, not diagnosed or treated, age, sex, marital status, occupation, education, monthly household income, body mass index, self-perception of overall health status.

Using the approach of the Andersen model [15], *individual health practices*, such as lifestyles related to diet, physical activity and consumption of tobacco and alcohol were considered the main explanatory variables (Fig 1) and conceptualized as modifiable health risk behaviors. The following four behaviors were used to assess patterns of modifiable health risk behaviors between two waves of the HWCS.

1. Diet was assessed using a semi-quantitative questionnaire, previously validated in the Mexican population [21]. The questionnaire included data on the frequency of consumption of 116 food items during the last 12 months. The WHO recommendations [22], established that adults should consume at least 600 g/day of fruits and vegetables to reduce health risk factors and avoid premature death. Therefore, *risk consumption* was defined accordingly as the consumption of < 3 portions/day of fruit and <4 portions/day of vegetables [23] and an *adequate consumption* as ≥3 portions/day of fruits and ≥4 portions/day of vegetables. Using that information, the following patterns of exposure to fruits and vegetables were established for the present analysis according to the behavior observed during the two waves of the study: a) maintain a risk consumption during both waves; b) maintain an adequate consumption during both waves (reference); c) change from a risk consumption to an adequate consumption and d) change from an adequate consumption to a risk consumption.
2. Physical activity (PA). A questionnaire validated and adapted for the Mexican population [24] was administered to collect data about leisure time physical activity over the past 12 months, including frequency, duration and intensity. The Compendium of Physical Activities [25] was used to identify the “METs” (metabolic equivalents) of each activity, and the MET/min for each activity was obtained using the formula: METs for the activity x time the activity was performed in minutes [26]. The WHO recommends that adults over 18 years old should perform ≥450 to ≤1800 METs/min/week of aerobic physical activity [27]. According to this measurement, *a risk level of physical activity* was defined as 0 to <450 and an *adequate level of physical activity* as ≥450. For the present analysis, the following patterns were generated based on the behavior during the two waves of the study: a) maintain a risk level of PA in both waves; b) maintain an adequate level of PA in both waves (reference), c) change from a risk level of PA to an adequate level; and d) change from an adequate level of PA to a risk level.
3. Tobacco consumption. Participants were asked if they had smoked 100 cigarettes or more in their entire life. Tobacco consumption was classified according to three categories: non-smoker (Never smoked a cigarette or smoked fewer than 100 cigarettes in entire lifetime), former smoker (having smoked at least 100 cigarettes in lifetime and having stopped smoking over one year ago) and active smoker (having smoked at least 100 cigarettes in lifetime and currently smokes) [28]. For this analysis, the following patterns were generated considering this behavior during the two waves of the study: a) active smoker in both waves; b) non-smoker or former smoker (reference) in both waves, c) change from active smoker to former smoker and d) change from non-smoker or former smoker to active smoker.
4. Alcohol Consumption. Participants were asked about the frequency of consumption of alcoholic beverages over the last 12 months. The average daily consumption of alcohol was calculated based on the Evaluation System for Nutritional Habits and Nutrient Consumption [29]. Participants were classified according to the risk level corresponding to daily alcohol consumption [30, 31] as: low-risk (1 to 40g/day for men and 1 to 20g/day for women), medium-risk (41 to 60g/day for men and 21 to 40g/day for women) and high-risk (>60g/day for men and >40g/day for women). For the present analysis, the following patterns were generated based on this behavior during the two waves of the study: a) maintain no/low-risk consumption (reference) in both waves, b) maintain medium/high-risk consumption in both waves, c) change from medium/high-risk to no/low-risk alcohol consumption and d) change from no/low-risk to medium/high-risk alcohol consumption.

### Covariates

Other explanatory variables include *individual characteristics* from the Andersen model [15]. *Predisposing characteristics*: age, sex, marital status, occupation and education. *Enabling characteristics*: monthly household income (quintiles). *Perceived needs*: self-rated health status (excellent, very good, good, average and poor). *Evaluated Needs*: chronic diseases diagnosed by a physician (none, at least one and two or more), accidents, injuries and surgeries during the last year (none and ≥ one), consumption of medications per week for those who reported no chronic diseases, accidents, injuries or surgeries (none, at least once and twice or more) and measurements of weight, height, blood pressure and the following laboratory measurements.

Venous blood samples were obtained from all participants after a minimum of 8 hours of fasting. These were processed according to standard criteria [32]. The results were classified as hypercholesterolemia (≥200 mg/dl), hypertriglyceridemia (≥150 mg/dl) [33], diabetes mellitus (≥126 mg/dl) [34], hyperuricemia (>6.8 mg/dl) [35], anemia (hematocrit <36% for women and <42% for men) [36] and chronic kidney disease (glomerular filtration rate < 60 ml/min/1.73m^2^) [37]. Blood pressure measurements were collected by trained nurses using an automatic digital monitor with the right arm supported at the level of the heart while the patient remain seated, after resting for five minutes. Levels of systolic blood pressure ≥140 mmHg and/or diastolic ≥90 mmHg was classified as hypertension [38]. Values out of range in the laboratory measurements and blood pressure during both stages of the study, for those who did not report such problems and did not take medication to treat them, was classified as none, one and two.

Measurement of anthropometric variables. Trained nurses carried out these measurements. Height was measured using a conventional stadiometer, without shoes and standing with the shoulders in a normal position. Weight was measured with a calibrated electronic scale (Tanita model BC-533), without shoes, standing and with minimal clothing. Body mass index (BMI) was calculated based on the formula *weight in kilos/(height in meters squared)* and classified according to WHO recommendations in: low weight <18.5, normal 18.50–24.99, overweight 25–29.99 and obese ≥30 [39].

### Analysis

The variables in this study were summarized according to the type of variable, either with central tendency and dispersion measures as well as proportions. The bivariate associations were assessed according to the distribution of each outcome variable (count of the utilization of each healthcare service) using Mann-Whitney U and Kruskal-Wallis tests. Changes in patterns of modifiable health risk behaviors between the two stages of the study were evaluated using McNemar tests. We tested the equidispersion assumption (mean equal to its variance) for each one of the response variables studied by using the dispersion index (VIT) as well as the asymptotic Böhning test. In all cases, we rejected the null hypothesis (p<0.01). As the outcome variables contained non-negative whole numbers (count data), equidispersion was analyzed with the asymptotic test, and showed that health services variables observed overdispersion with the exception of preventative health services (p<0.05). Consequently, we decided to use zero-inflated negative binomial regression models. Therefore, for the frequency of utilization of each healthcare service, zero-inflated negative binomial (ZINB) regression models were adjusted with robust estimators of the standard errors because some of the participants belong to the same family and workplace. The same procedure was conducted with the main independent variables and covariates, including changes among the two stages of the study: alcohol consumption, tobacco consumption, leisure time physical activity, consumption of fruits and consumption of vegetables. These models estimated the exponential of Beta (e^B^) for each variable. This transformation can be interpreted as the average change in each category versus the reference, adjusted for covariates. All associations with an alpha of 0.05 were considered significant; the adjustment variables were included as footnote in tables that required it. All the analyses were performed using STATA 14 (Stata Corporation, College Station, TX, USA).

### Ethical considerations

The original study protocol was approved by the Ethics and Research Commissions from the IMSS and the National Institute of Public Health in Mexico. All the participants provided written informed consent.

## Results

**Table 1** shows the overall characteristics of the sample during the second stage of the study. The mean age of the participants was 54.5 years old and 69.8% were women. **Table 2** shows the prevalence of the lifestyle variables during the two stages of the study. A decrease of 4.3 percentage points (p<0.001) in the prevalence of *active smokers* was observed, and also an increase of 5.7 percentage points (p = 0.015) was seen in the prevalence of *risk level of physical activity*. The changes observed in consumption of fruits, vegetables and alcohol were not statistically significant.

**Table 1:** General sample characteristics and health conditions (2010–2013), Health Workers Cohort Study, IMSS, (n = 698).

	(%, mean or median)
**Variables**	
**Age (mean)**	54.5 years (IR:45–64)
**Sex (women)**	69.8
**Marital Status**	
Married-free union	63.2
Widowed-separated-divorced	22.4
Single	14.5
**Occupation: **	
Retired	24.6
Professional employees	45.1
Non-professional employees	21.8
Unemployed-student-homemaker	6.6
**Education: **	
Elementary	14.9
Secondary-high school	38.5
College-University** **	46.6
**Monthly household income (median)**	888.4* (IR: 484.6–1,615.3)
**Body Mass Index**	
Overweight	39.8
Obesity	17.4
**Diabetes**	5.6
**Hypertension**	16.6
**Hypercholesterolemia**	16.9

**Table 2:** Prevalence of modifiable health risk behaviors and its change among two stages of the Health Workers Cohort Study (HWCS) 2004 and 2010.

Modifiable health risk behaviors	2004	2010				McNemar Test
	Exposed (CI 95%)	Discontinued exposure (CI 95%)	Continued exposure (CI 95%)	Began exposure (CI 95%)	Exposed (CI95%)	
Active smoker	15.9 (13.2–18.6)	- 6.9 (5–8.8)	= 9 (6.9–11.2)	+ 2.6 (1.4–3.8)	= 11.6 (9.2–14)	<0.001
<3 portions of fruit/day	46.7 (43–50.4)	- 16 (13.3–18.8)	= 30.7 (27.2–34.1)	+ 17.3 (14.5–20.2)	= 48(44.3–51.7)	0.600
<4 portions of vegetables/day	88.5 (86.2–90.9)	- 10.3 (8.1–12.6)	= 78.2 (75.2–81.3)	+ 7.7 (5.7–9.7)	= 86 (83.4–88.5)	0.130
Physical activity: <450Met/min/week	52.9 (46.6–59.1)	- 15.6 (12.9–18.3)	= 37.2 (33.7–40.8)	+ 21.3 (18.3–24.4)	= 58.6 (52–65.2)	0.015
Alcohol: medium-high-risk	3.3 (1.5–5.1)	- 2 (1–3)	= 1.3 (0.5–2.1)	+ 1.7 (0.8–2.7)	= 3 (1.3–4.7)	0.845

Data about the utilization of healthcare services during the previous 12 months showed that 82.2% did not use emergency room services, 61.6% did not use preventive services, 58.2% did not visit a specialist, and 32.1% did not visit a general practice physician. The average use of these services during the previous 12 months was 1.4, 1.3, 2 and 2.7 times, respectively. **Table 3** shows the bivariate associations for the four healthcare services variables. Significant differences were observed in the number of preventative consultations (p = 0.002) and general practice consultations (p = 0.014) for the fruit consumption categories, and among the physical activity categories and number of preventative consultations (p = 0.001).

**Table 3:** Bivariate associations of number of consultations of each healthcare service, in the Health Workers Cohort Study (HWCS), 2004 and 2010.

Explanatory variables for the utilization of healthcare services	preventative medicine*	Emergency room services *	Medical specialists *	General Practice *
† Tobacco consumption 2004 and 2010	0.068	0.441	0.260	0.673
† Fruit consumption 2004 and 2010	0.002	0.497	0.879	0.014
† Vegetable consumption 2004 and 2010	0.752	0.420	0.632	0.446
† Physical activity in METs/min/week 2004 and 2010	0.001	0.420	0.239	0.183
† Alcohol consumption 2004 and 2010	0.528	0.140	0.418	0.236
† Chronic diseases 2010	<0.001	<0.001	<0.001	<0.001
‡ Surgeries or injuries 2010	0.256	0.005	0.001	0.241
† Ongoing consumption of medications 2010	0.320	0.662	0.792	0.411
† Hyperuricemia, hyperlipidemia, hyperglycemia, chronic kidney disease, anemia and high blood pressure, not diagnosed or treated. 2010	0.124	0.017	0.006	0.061
† Age 2010	0.116	0.905	0.007	<0.001
‡ Sex 2010	0.136	0.917	<0.001	<0.001
† Marital status 2010 (and imputed from 2004)	0.275	0.301	0.007	<0.001
† Occupation 2010 (and imputed from 2004)	0.312	0.274	0.020	0.012
† Education 2010 (and imputed from 2004)	0.037	0.122	0.352	<0.001
† Monthly household income (quintiles)	0.021	0.026	0.383	0.068
† Body mass index 2010	0.656	0.432	0.331	0.122
† Self-perception of overall health status 2010	0.199	<0.001	0.002	<0.001

† Kruskal–Wallis Test.

‡ Wilcoxon-Mann-Whitney Test.

* p values for each test are shown in the table. Each test compared the distribution of the number of consultations (for each healthcare service) between the groups defined of each factor (using the categories described in methods).

**Table 4** presents the ZINB models for each healthcare service used. In terms of associations with modifiable health risk behaviors (Fig 1), the principal findings are as follows: I) Preventative consultations: a) 49% higher in the pattern *change from active smoker to former smoker* compared to *be non-smoker/former smoker* in both waves (e^B^: 1.49; CI95%: 1.06–2.10); b) 27% lower for the category *keep a risk consumption of fruits in both waves* compared to the pattern *keep an adequate consumption of fruits in both waves* (e^B^: 0.73; CI95%: 0.54–0.98); c) 27% lower for the pattern *keep a risk level of PA* in *both waves* (e^B^: 0.73; CI95%: 0.56–0.94) and 39% lower for the pattern *change from adequate level of PA to risk* in the two waves (e^B^: 0.61; CI95%: 0.43–0.85), as compared to *adequate physical activity* in both two waves, II) General practice consultations were 45% lower for the pattern *change from medium/high-risk* to *no/low-risk consumption of alcohol* compared to the category *no/low-risk consumption of alcohol in both waves* (e^B^: 0.55; CI95%: 0.32–0.95), and III) Medical specialist consultations were 44% higher for the category *change from active smoker* to *former smoker* compared to the pattern *non-smoker or former smoker in both waves* (e^B^: 1.44; CI95%: 1.004–2.07; p = 0.048).

**Table 4:** Zero-inflated negative binomial regression models for the number of consultations of each healthcare service; Health Workers Cohort Study (HWCS) 2004 and 2010.

	Preventative medicine (n = 748)			Emergency room services (n = 731)			General practice (n = 769)			Medical Specialist (n = 755)		
	e^B^	SE	p	e^B^	SE	p	e^B^	SE	p	e^B^	SE	p
**Patterns of tobacco consumption between 2004 and 2010 (Ref: non-smoker or former smoker in both waves)**												
Risk consumption in both waves	1.15	0.31	0.613	0.57	0.49	0.514	1.19	0.16	0.206	0.59	0.18	0.090**
Change low risk to active smoker	0.70	0.16	0.123	0.55	0.22	0.130	1.12	0.15	0.390	0.81	0.17	0.328
Change active smoker to former smoker	1.49	0.26	0.021*	1.00	0.35	0.993	1.16	0.13	0.182	1.44	0.27	0.048*
**Patterns of consumption of fruits (Ref: ≥3 portions/day during both waves)**												
Risk consumption in both waves	0.73	0.11	0.035*	1.03	0.23	0.896	0.97	0.08	0.691	1.04	0.14	0.789
Change from risk to low risk	0.83	0.13	0.240	1.01	0.28	0.976	1.16	0.12	0.166	0.89	0.16	0.511
Change from low risk to risk	1.10	0.14	0.471	0.73	0.22	0.293	1.15	0.10	0.100	0.95	0.13	0.734
**Patterns of consumption of vegetables (Ref: ≥4 portions/day during both waves)**												
Risk consumption in both waves	1.55	0.48	0.150	2.24	1.59	0.254	1.11	0.19	0.540	0.98	0.26	0.953
Change from risk to low risk	1.31	0.43	0.419	1.61	1.23	0.533	1.19	0.23	0.370	0.84	0.25	0.559
Change from low risk to risk	1.37	0.48	0.372	3.30	2.40	0.100	1.21	0.24	0.344	1.32	0.40	0.362
**Patterns of physical activity in METs/min/week (Ref: adequate physical activity in both waves)**												
Risk consumption in both waves	0.73	0.09	0.013*	1.06	0.23	0.794	0.97	0.08	0.703	1.06	0.14	0.650
Change from risk to low risk	0.81	0.12	0.148	0.72	0.21	0.249	1.04	0.09	0.679	1.20	0.18	0.217
Change from low risk to risk	0.61	0.11	0.004*	0.91	0.25	0.728	0.90	0.09	0.296	0.86	0.15	0.396
**Patterns of alcohol consumptions (Ref: no/low-risk consumption in both waves)**												
Risk consumption in both waves	1.75	0.66	0.140	NE†	NE†	NE†	1.17	0.29	0.521	1.61	0.49	0.116
Change from low risk to high risk	0.45	0.22	0.103	1.91	1.02	0.229	1.39	0.26	0.081**	0.64	0.38	0.447
Change from high risk to low risk	0.77	0.38	0.601	NE†	NE†	NE†	0.55	0.15	0.031*	1.32	0.56	0.505
**Chronic diseases (Ref: none)**												
One	0.99	0.18	0.946	2.16	1.49	0.262	1.50	0.27	0.028*	1.45	0.43	0.210
≥ Two	1.53	0.26	0.013*	2.63	1.32	0.056	2.16	0.36	<0.001*	1.39	0.39	0.243
**Surgeries or injuries (Ref: none)**												
≥One	0.77	0.25	0.434	3.01	1.21	0.006*	1.23	0.17	0.119	1.69	0.36	0.014*
**Ongoing consumption of medication (Ref:none)**												
One	0.98	0.30	0.960	1.06	0.52	0.910	1.66	0.32	0.008*	1.64	0.46	0.082**
≥Two	0.81	0.38	0.654	2.38	1.20	0.087**	2.65	0.68	<0.001*	1.29	0.47	0.482
**Hyperuricemia, hyperlipidemia, hyperglycemia, chronic kidney disease, anemia and high blood pressure, not diagnosed or treated.(Ref. none)**												
One	0.79	0.10	0.054**	0.61	0.14	0.029*	0.95	0.08	0.574	0.72	0.10	0.014*
Two	0.79	0.30	0.527	1.15	0.45	0.730	0.69	0.17	0.134	1.27	0.33	0.357
**Age (Ref: ≥20 to ≤39)**												
≥40 to ≤49 years	1.15	0.24	0.489	0.79	0.24	0.426	0.98	0.13	0.900	0.85	0.19	0.465
≥50 to ≤59 years	1.24	0.26	0.308	0.77	0.23	0.393	1.14	0.16	0.346	0.86	0.18	0.470
≥60 to ≤69 years	1.42	0.31	0.107	0.57	0.23	0.162	1.26	0.19	0.111	0.83	0.19	0.411
≥70 years	1.44	0.38	0.163	0.59	0.24	0.186	1.31	0.22	0.113	0.88	0.23	0.639
**Male (Ref: Female)**	1.03	0.20	0.873	1.27	0.28	0.276	0.89	0.08	0.220	0.73	0.10	0.026*
**Marital Status (Ref:single)**												
Married-Free Union	0.95	0.17	0.790	0.95	0.28	0.876	1.06	0.11	0.574	0.90	0.16	0.552
Widowed	1.12	0.25	0.609	0.88	0.36	0.767	1.05	0.14	0.748	1.31	0.30	0.233
Separated-Divorced	1.22	0.26	0.350	1.62	0.54	0.145	1.22	0.16	0.113	0.87	0.19	0.520
**Occupation (Ref:retired)**												
Professional	1.19	0.16	0.186	0.85	0.22	0.534	0.92	0.08	0.340	0.92	0.13	0.554
Non-professional employment	1.32	0.25	0.136	1.13	0.34	0.697	0.99	0.11	0.906	0.91	0.17	0.595
Unemployed/Student/Homemaker	0.84	0.22	0.489	0.82	0.33	0.627	0.74	0.14	0.101	0.44	0.13	0.006*
Other	0.66	0.26	0.293	0.00	0.00	<0.001*	0.91	0.18	0.617	0.78	0.40	0.629
**Education (Ref:elementary)**												
Middle School	0.97	0.22	0.901	0.56	0.20	0.101	0.93	0.11	0.544	1.17	0.23	0.430
High School	1.39	0.32	0.155	0.77	0.24	0.398	0.97	0.11	0.765	1.26	0.25	0.256
College-University	1.45	0.30	0.071**	0.53	0.17	0.053**	0.96	0.11	0.752	1.32	0.26	0.158
**Monthly Household Income Quintiles (Ref:first quintile)**												
2°	0.64	0.13	0.031*	1.11	0.28	0.682	0.99	0.10	0.904	0.93	0.17	0.674
3°	0.91	0.16	0.611	0.78	0.23	0.392	1.06	0.12	0.611	0.98	0.17	0.914
4°	0.98	0.19	0.896	0.88	0.32	0.732	0.89	0.10	0.307	0.88	0.16	0.491
5°	0.84	0.16	0.379	0.74	0.27	0.410	0.98	0.13	0.854	0.81	0.18	0.344
**Body Mass Index (Ref: normal > = 18.5 - <25)**												
Low weight<18.5	NE†	NE†	NE†	NE†	NE†	NE†	1.54	0.29	0.022*	2.41	0.74	0.004*
Overweight ≥25 - <30	0.83	0.10	0.119	0.95	0.19	0.805	0.91	0.07	0.216	0.94	0.11	0.580
Obese ≥30	0.82	0.11	0.143	0.63	0.16	0.064**	0.97	0.08	0.725	0.84	0.13	0.248
**Self-perception of overall health status (Ref: excellent)**												
Very good	0.75	0.21	0.299	0.68	0.36	0.460	1.84	0.44	0.010*	0.64	0.25	0.241
Good	0.65	0.17	0.090**	0.70	0.33	0.461	2.01	0.45	0.002*	0.67	0.25	0.292
Average	0.73	0.19	0.237	1.11	0.53	0.830	2.47	0.54	<0.001*	0.76	0.28	0.454
Poor	0.74	0.27	0.408	1.54	0.89	0.460	2.64	0.64	<0.001*	1.19	0.49	0.676
Don’t know	0.18	0.16	0.058**	2.47	2.05	0.276	1.60	0.74	0.308	0.85	0.57	0.808
**Inflated** § ***												
**Chronic diseases (Ref:none)**												
One				12.94	1.73	<0.001*	-0.5	0.52	0.298	-0.3	0.49	0.432
≥ Two				10.31	11.90	0.386	-1.4	0.44	0.001*	-1.5	0.68	0.020*
**Hyperuricemia, hyperlipidemia, hyperglycemia, chronic kidney disease, anemia and high blood pressure, not diagnosed or treated.(Ref. none)**												
One							0.65	0.32	0.045*			
Two							-0.4	1.83	0.791			
**Male (Ref:Female)**	14.66	1.41	<0.001*									
**Health status self-perceived (Ref:excellent)**												
Very good	-19.8	3.56	<0.001*									
Good	-0.17	3.55	0.961									
Average	0.21	3.51	0.953									
Poor	-29.3	3.57	<0.001*									
Don’t Know	-18.7	3.52	<0.001*									

**Models adjusted by the following variables:** tobacco consumption, consumption of fruits, consumption of vegetables, alcohol consumption, leisure time physical activity, chronic diseases, surgeries or injuries, ongoing consumption of medications, hyperuricemia, hyperlipidemia, hyperglycemia, chronic kidney disease, anemia and high blood pressure, not diagnosed or treated, age, sex, marital status, occupation, education, monthly household income, body mass index, self-perception of overall health status.

e^B^: beta exponential of the model. SE: Robust standard error.

*p ≤ 0.05.

**p ≤ 0.05

***p ≤ 0.10.

Preventative medicine: inflated (sex, self-perception of health status).

§Emergency room services: inflated (chronic diseases). General practice: inflated (chronic diseases, hyperuricemia-hyperlipidemia-hyperglycemia-chronic kidney disease-anemia-high blood pressure). Medical Specialist: inflated (chronic diseases).

†Not possible to estimate because of lack of variability, these categories have few individuals and none had consultations.

In addition, other variables that were determinants of the utilization of healthcare services were (**Table 4**):

- Predisposing characteristics: Men and unemployed/students/homemakers tended to have fewer medical specialists consultations.
- Enabling characteristics: Participants in the 2^nd^ income quintile had fewer preventative consultations.
- Perceived needs: A self-perceived poor health status was associated with more visits to a general practice physician.
- Evaluated needs: Report of one chronic disease was associated with more consultations with a general practice physician and a report of two or more chronic conditions was associated with more preventative consultations and visits to a general practice physician. Report of one or more surgeries or injuries was associated with a higher number of emergency room visits and consultations with a specialist. Ongoing consumption of a medication and consumption of two or more medications was associated with more visits to a general practice physician. Laboratory and blood pressure measurements with values out of range (as previously classified) was associated with fewer emergency room visits and consultations with a specialist. Participants with measurements of low weight were associated with more consultations with a general practice physician and specialists.

The variables that were determinants of the utilization of healthcare services associated with the inflated term were **(Table 4)**: report of one chronic diseases for emergency room services (p<0.001); two or more chronic conditions for medical specialist (p = 0.02) and general practice physician (p = 0.001); laboratory or blood pressure measurements out of range (as previously classified), for general practice physician (p = 0.045); male sex (p<0.001) and very good or poor self-perception of health status (p<0.001) for preventative consultations.

## Discussion

The present study identified an association between prospective patterns of modifiable health risk behaviors and its change over time with the utilization of healthcare services. These results may establish a precedent that has not been widely explored about modifiable health risk behaviors and health services use in the context of a social security institution providing health insurance and services for preventative and treatment, and a package of other comprehensive benefits; and belonging to a upper-middle income country experiencing health reform and high fragmentation of its healthcare system, as it is Mexico [40]. These results show that changes in specific MHRB are associated with the utilization of health care services provided at IMSS healthcare facilities. In addition, this information has been assessed among health workers (medical staff, administrative, and academic employees and their families), who, because of their proximity to healthcare services, would have a greater knowledge and awareness of the importance of keeping health behaviors as healthy possible. Therefore, seeking this kind of association in a follow-up study in countries like Mexico where there are still limitations in carrying out this type of analysis is a novel approach and consistent with other studies which have previously explored these relationships.

In terms of tobacco consumption, a higher number of consultations with specialists or preventative consultations among participants who change their behaviors from being active smokers to former smokers is similar to a report by Wacker, M. [13]. These results may indicate that former smokers have a higher probability of receiving treatment involving hospitalization, rehabilitation and consumption of medications. This may be due to a health problem that led to smoking cessation as well as the use of more services [13]. On the other hand, other authors have not found an association between tobacco consumption and the long-term use of medical services among adults aged 65 years or more [41].

In terms of diet, there were fewer preventative consultations by participants who maintain a consumption of <3 portions/day of fruits, which was similar to the results reported by Jahangir, E. [42], but are different from the findings observed by Yen-ju, L. [11] who found that persons ≥12 years old who consumed either fruits, milk, meat or grains daily were less likely to use outpatient services.

In terms of physical activity, subjects who maintained the risk level pattern of physical activity among the two waves or who changed from an adequate to a risk level had fewer preventative consultations. However, these results are not consistent with findings from other authors, who observed a lower use of healthcare services among persons who practice physical activity [12, 14, 42]. On the other hand, our results related to a lower use of preventative services associated with a low consumption of fruits or a low level of physical activity may be associated with an unhealthy lifestyle pattern, for example being less aware of the importance of using healthcare services [11].

In terms of alcohol consumption, there were fewer general practice visits by patients who change from a medium/high-risk to no/low-risk alcohol consumption pattern, which is different than the results observed by Zarkin, G. [14], who found that all alcohol consumption patterns are associated with a lower use of health services. In addition, our results are not consistent with findings by Jahangir, E. [42], who observed a lower use of preventative services among those with heavy drinking. The difference observed between studies might be related to different criteria of classification of alcohol consumption categories [14]. Meanwhile, other authors have found no evidence of an association between alcohol consumption and the use of health services among persons 25 to 74 years old [13], or between alcohol consumption and the long-term care use by adults ≥65 years old [41].

The dose-response relationship between poor self-perceived health and increased visits with general practice physicians is similar to other studies [43]. For example, it has been observed that persons who are 50 years or older with a poorer quality of life (including self-perception of health status) were more likely to use outpatient health services.

Our findings about other variables such as sex, report of chronic conditions and education, were generally consistent with the findings reported previously in the literature [11, 13, 41–43].

The present study found that participants who used fewer preventative medicine services were those who had a low consumption of fruits or a low level of physical activity, whereas participants who quit smoking used more preventative or specialist services; the consequences of these changes of behaviors should be subject to future researches.

Given the association between modifiable health risk behaviors and an increased risk of developing NCD [2], its relationship with the healthcare services utilization seems evident. Therefore, the revision of clinical records from HWCS participants to determine the diagnosis of NCD and their health service utilization at IMSS is an important task for future analysis. Nevertheless and to deal with this limitation, the associations estimated with modifiable health risk behaviors were adjusted for the presence of NCD, thus the results of this study about the relationship among lifestyles behaviors and use of healthcare and preventative services is independent of health conditions. These findings suggests that lifestyle behaviors by themselves affect the use of services. Thus, it is important to emphasize that among this relationship, a bidirectional association from; a) *utilization of health services* ***→*** *MHRB* [8, 9] and b) *MHRB* ***→*** *utilization of healthcare services* [10–13] could be observed.

In addition, other known determinants common to both lifestyles and the healthcare services utilization were not measured. In terms of the relationship between *MHRB****→*** *utilization of healthcare services*, this could be explained as a protector or as a risk factor due to the impact of lifestyles on health status, in the context of different individual vulnerabilities [17], as well as by factors that are related with health promotion actions [16]. However, the decision to seek medical care depends on individual perceptions of one’s health problems [15].

While it is worth mentioning the progressive increase in the number of consultations with general/family practice physicians as the perception of one’s health status worsens. Since this relationship is independent of the objective health status and MHRB, it would reflect the influence of one’s subjective health status on the demand of services, which only occurs in general/family medicine since the use of this service is immediate and depends on the user requesting care. While the relationship between subjective and objective health status is complex, self-perception of health status has been shown to be associated with the latter [44].

Lastly, more investigations need to be conducted in Mexico about the determinants of healthcare services utilization, in order to identify opportunities to increase the coverage of prevention and health promotion actions within the health system. The present study is contributing to that approach identifying differences in the utilization of healthcare services associated with certain lifestyles and its change over time, which has not been widely explored. Evaluating the use of services by populations that shift to a different pattern of MHRB is thus shown to be relevant, in order to identify how this affects health using data of a cohort study.

**Limitations of the study**: Given that only two measurements were collected over a 6-year period, it was not possible to identify the fluctuations in the follow-up period, including changes in lifestyle or in the use of health services over time, which are mostly dynamic and iterative phenomena. In addition, even though the determinants of the use of health services may include other variables that were not measured, such as contextual characteristics as well as other individual characteristics, social networks, health beliefs, transportation time and waiting time to receive care, user satisfaction and pregnancy [15]. Although the available information had limitations, we believe the model proposed enables us to define certain aspects involved in the relationship between lifestyles and the use of services that have not been explored previously in Mexico.

Meanwhile, all participants of the HWCS are affiliated with IMSS, and there is the possibility of moral hazard [45], that is, the impact of the affiliation with IMSS on fewer preventative actions and the over-consultation of medical services, which could not be measured. The results could also be confounded by health conditions that were only partially measured. Lastly, information was not available about the reasons for utilizing the healthcare service.

**Fig 2:**
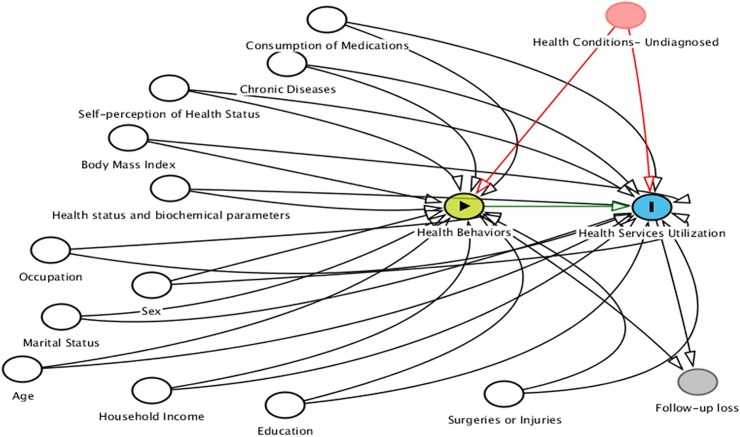
Directed Acyclic Graph of the associations explored in this study.

Given the lack of a follow-up measurement of the use of services, variables were created to determine the change in lifestyles over the follow-up period in order to explore its influence on the current use of services. Nevertheless, a causal relationship could not be defined between the use of health services and its associated factors due to the inability to establish the temporality and the direction of the association. It is worth mentioning that it is methodologically challenging to establish this association since the relationship between lifestyles and the use of services is iterative, dynamic and bi-directional. Likewise, other possible patterns of association and new health conditions could be observed in this study (even before being diagnosed or not diagnosed). Both could determine changes in the patterns of health services utilization and those in turn explain some of the changes in health behaviors observed in this population. Therefore, the following relationships might be possible:

***B*** (health behaviors) —***> C*** (health services utilization), but also another possible pathway would be observed:

***A*** (health conditions- undiagnosed and therefore not adjusted for-) —***>B*** (health behaviors) —***> C*** (health services utilization), with another arrow from ***A to C***,

These associations can be considered a classic phenomenon of confounding factors, since health conditions are common causes of both health behaviors and health services utilization. Finally, we could also have another possible pathway:

***A*** (health conditions- undiagnosed and therefore not adjusted for-) —***> C*** (health services utilization) —***>B*** (health behaviors). This one is known as reverse causality, but after we use the first pathway as our reference. In this study we were not able to consider all these pathways due to limitations in measuring health conditions (subclinical or undiagnosed) between waves. We believe that these mixed effects are very difficult to identify in this study and thus we recognized this as an important limitation of our study. See Directed Acyclic Graph. Fig 2

It was also not possible to evaluate potential selection bias due to loss of follow-up, because HWCS participants in the 2004–2006 stage were invited to be part of the ongoing stage in 2010–2012, but not all participants continued to the next stage and the study methods and funding for collecting data for that stage were designed and obtained to carry out the follow-up measurement in a sample of these participants. In addition, and since the original study design was not intended for a probabilistic sampling, external validity was limited to subjects having the characteristics of the final analytical sample. Given the nature of self-reporting used in the study, memory bias was possible, which could result in an over- or under-reporting of information.

In addition, our results have some important limitations. For instance, we could not consider some important confounder variables such as other Social Determinants of Health (SDH). Also, the directionality of the relationships studied were not clearly established, not just because of the iterative and dynamic nature of healthcare utilization, but also due to insufficient data. Finally, contextual effects were not estimated in this analysis. However, we could reasonably expect that the participants of this study share some social determinants as they come from a same context. It is important to point out that all the participants in this study were health workers at the same workplace, The Mexican Social Security Institute. So, they do share at least one social context. Certainly, they must live in different social conditions, but at least we could adjust our analysis for the main social factors at individual level such as: education, occupation and income which are also considered SDH by the World Health Organization in the Americas Region [46].

**Strengths of the study**: The study used prospective data related to health-related lifestyles as the principal exposures of interest. Unlike strictly cross-sectional studies, this enabled incorporating the lifestyles, considering that the behavior was kept or was changed over time. The ZINB models used in the study made it possible to address excess zeros (structural and sampling) in the response variables as well as unobserved heterogeneity. The models were adjusted by the most relevant covariates, known by the Anderson model, to be determinants of the use of services. These primarily included four lifestyle determinants and the presence of chronic diseases. Thus, the associations with lifestyles that were found were independent and necessarily related to other causal routes. Lastly, unlike previous studies, this work evaluated associations with the use of different health services (preventative medicine, emergency room services, family/general practice and specialist physicians), making it possible to identify the heterogeneity of the effect of lifestyles on different healthcare services utilization.

## Conclusions

Certain modifiable health risk behaviors are associated with fewer preventative consultations. These MHRB include either maintaining consumption or having an insufficient consumption of fruits, having an insufficient level of PA, or changing from an adequate to an insufficient level of PA. In addition, reducing alcohol consumption and quitting smoking are associated with fewer consultations with family/general practice and specialist physicians, respectively. These results suggest that not only the socio-demographic characteristics but also their individual behaviors influence the use of services.

The finding that participants exposed to certain prospective patterns of modifiable health risk behaviors use fewer preventative medicine consultations is relevant, and suggest that this population does not benefit from preventative practices. It is also important to highlight that the participants who changed from active smoker to former smoker had more consultations with specialists, which could be related to already-established health problems and which currently represents a greater economic burden for the institution.

Based on our results, we consider it necessary to identify the consequences of the differential use of health services associated with MHRB and strengthen interventions at the level of non-personnel services with a public health approach in order to create healthier lifestyles in the population that would otherwise not use the services.
